# Feedback in an Epidemic?

**DOI:** 10.7759/cureus.22008

**Published:** 2022-02-08

**Authors:** Amr Taha

**Affiliations:** 1 Oral and Maxillofacial Surgery, Hull University Teaching Hospitals NHS Trust, Hull, GBR; 2 Paediatric and Special Care Dentistry, City Health Care Partnership (CHCP) Dental Services, Hull, GBR

**Keywords:** covid-19 medical education, dental-education, teaching feedback, dentistry related articles, clinical dentistry, clinical feedback

## Abstract

Undergraduates and postgraduates frequently receive feedback on their clinical and non-clinical performance and progression throughout their studies and career. Good quality feedback has been shown to improve trainees’ confidence and performance. This article discusses the benefits of feedback and reviews the communication, technical, financial, and networking barriers to feedback introduced by coronavirus disease 2019 (COVID-19) and its impact on the quality of medical and dental education in the UK, followed by a critical reflection. In addition, it reviews the pros and cons of self-assessment of clinical learning, and it provides an overview of the most widely accepted feedback models: Pendleton’s rules, SET-GO method, agenda-led, outcome-based analysis (ALOBA) model and Prepare to Ask-Discuss-Ask-Plan Together (Prepare to ADAPT) on the quality of feedback received. The aim is to identify the most suitable feedback method to help trainees with their clinical and professional development during the COVID-19 pandemic and any possible pandemics in the future.

## Introduction and background

Due to the introduction of additional coronavirus disease 2019 (COVID-19) barriers, there has been a lack of build-up of a positive direct relationship between trainer and trainee, and this has had a negative effect on constructive feedback acceptance [1]. Receiving feedback helps trainees to improve their technique, confidence and performance [2-3]. Self-assessment is a powerful learning tool allowing trainees to monitor their performance in a way that reduces dependence on their trainers [4]. Structured discussion followed by interactive feedback encourages self-evaluation, reducing trainee anxiety and stress [5-6].

This article aims to discuss the benefits of feedback before the COVID-19 pandemic and reviews the communication, technical, financial, and networking barriers’ effects on feedback received by medical and dental trainees during the COVID-19 pandemic. It assesses the impact on the quality of medical and dental education in the UK by a critical reflection over the last two years spent as a dental core trainee during the pandemic. It also provides an overview of the most widely accepted feedback models: Pendleton’s rules, SET-GO method, agenda-led, outcome-based analysis (ALOBA) model, and Prepare to Ask-Discuss-Ask-Plan Together (Prepare to ADAPT) on the quality of feedback received.

PubMed and the Cochrane library were used as the search engine and the phrase “Clinical feedback for trainees’” was used. The search dates were between 1985 and 2022. A total of 1766 articles were found by the search. Systematic review and review articles in English were then selected and duplicates removed, and the remaining articles on the feedback models listed above were reviewed.

## Review

Benefits of feedback

Receiving feedback is one of the most crucial learning tools helping trainees reach their utmost learning potential and accelerating their training progression [7]. Consistent and successful feedback helps to reinforce good practice and propel the learner towards the desired learning goals. The direct observation of the trainee’s performance is very important to tailor the feedback they receive, making it more effective in order to correct the poor performance [8]. Delivering this feedback directly helps the trainee better understand their development at that specific learning point [9-10]. As the feedback is a creative discussion between the trainer and trainee, it allows them to discuss the performance of the trainee in a friendly atmosphere, helping the trainee to make a suitable decision to reach the planned outcome aiding their learning progression [11]. Furthermore, the appropriate learning environment helps trainees develop an improved perception of their current skills and plan for future learning requirements [11]. However, if the trainers try to save time by not giving feedback to their trainees, it can lead to a negative effect on their performance [12].

The educational programme should be intentionally designed to help motivate the trainee to engage deeply in receiving and giving feedback [11]. Feedback should not be judgemental to avoid a feeling of embarrassment in the trainee, leading to them being defensive, and it should be descriptive [13]. The duration of feedback should be suitable for discussing the performance of the trainee, guidance, and planning for the potential of the outcome. Following the establishment of mutual trust, feedback should be in a confidential location with a clearly defined outcome. The feedback should be accurate and balanced for it to be beneficial for the trainee [14]. A study by Cantillon P and Sargeant J, 2008, found that feedback is best accepted in shorter rather than longer discussions [8].

Well-constructed feedback improves the trainees' clinical practice and helps to support their self-reflection, aiding them in working towards their learning objectives [15]. Trainees must be familiar with their assessment criteria to better understand the context in which their feedback is given, helping in their clinical development [16]. Burgees A and Mellis C (2015) suggested the following principles for beneficial feedback: honest, planned, considering the place, timing and environment, focused on behaviour (not personality), verified by the recipient, concise, specific, descriptive and explicit [15].

A comparison between trainees who received feedback and those who did not receive feedback demonstrated that the group that received feedback improved their technique. In addition, this group found that the feedback was accurate and useful [2]. Furthermore, a separate study was carried out by Spanager et al. (2015) targeting the feedback for a group of trainees via reviewing a post-procedure video. It showed an improvement in the trainees’ confidence and improved their performance [3]. Proving feedback and aiding and observing a trainees’ development has other advantages; it helps boost the supervisors’ sense of job fulfilment and adds to their personal development [17].

In general, more detailed, and specific feedback is likely to be more beneficial than very vague and general feedback. Also, the complexity and/or the length of the comment can affect the feedback. Too long or too complex feedback may confuse the trainee making it useless. In addition, lengthy feedback may dilute the message the trainer is trying to get across [18]. The complexity of the feedback affects the outcome of learning and the ability of the trainee [19].

The difficulties with giving feedback lie with the trainers not wishing to give negative feedback to keep a good relationship with their trainees as some trainees may feel apprehensive about receiving negative feedback [20]. 

COVID-19 and feedback

The COVID-19 pandemic has had a drastic effect on medical and dental education throughout the world. Consequently, the quality of feedback has been negatively affected due to the unintentional introduction of multiple layers of barriers and social distancing affecting both verbal and non-verbal communication. Multiple examples of these barriers are apparent in the vast majority of workplaces and have affected the relationships of trainers and trainees. For example, social gatherings have always served as a platform for friendly interactions in a more comfortable and less stressful setting. The successful social distancing between the trainer and trainee has reduced interactions and prevented the build-up of a positive relationship, which has proven to have a negative impact on constructive feedback acceptance [1].

Constantly, wearing facial coverings has significantly reduced non-verbal feedback. FFP3 respirator masks frequently worn in clinical medical and dental settings make it very difficult for both trainer and trainee to hear and speak to each other. This has reduced the effectiveness of verbal communication and, therefore, negatively impacted clinical teaching opportunities. Furthermore, the move from a face-to-face platform to a virtual platform has introduced many additional technical and financial barriers. The lecture-based teaching in both large and small groups has now become dependent on the trainer and trainee's ability to source or self-fund and learn to use multiple digital learning equipment and online platforms for virtual meetings.

The move to virtual platforms has also transitioned across to feedback with more trainers utilizing online feedback platforms, such are E-portfolios, for significant learning events. This type of feedback is often completed days to weeks following the educational encounter. Instead of being individual and personalised, it is often generalised and less helpful to the trainees’ development. A study of students receiving their annual review of competence progression (ARCP) feedback virtually using online video conferencing platforms, such as Microsoft Teams and Zoom, demonstrated less effective communication than in face-to-face meetings with their trainers. However, they acknowledged that virtual meetings were more convenient, and this reduced travel time and saved commuting fees [21].

The advantages of virtual meetings were also recognized by UK trainers and panel members, as they often had to travel several hours to attend an ARCP meeting in different training hospitals that can be considerably far from one another [22]. The reduction in the use of motor vehicles for commuting over time will consequentially have a beneficial effect on the environment by reducing the carbon footprint.

In addition to the reduced quality of feedback medical and dental trainees may be receiving during the COVID-19 pandemic, all hands-on courses and regional face-to-face training have been cancelled. These sessions were usually an opportunity for lecturers and trainers from multiple regions to socialize and share their expertise, acting as a fountain of knowledge to the trainees. Moreover, not being able to meet in person can have a negative effect on both the trainees' and the trainers' state of mind and hinder their ability to catch up on the latest professional updates [23].

Self-assessment

One side of the feedback is the trainee’s self-assessment. This helps the trainees to engage actively in the process to achieve their goals by monitoring their actions, which helps them progress towards their goals [9,24]. To improve the performance of the trainee, the trainer’s assessment and trainee's self-assessment are shared. Then the plan is set up for future performance improvement [25]. Trainees’ performance can be negatively impacted by the lack of feedback from their trainers, as they may inappropriately assess themselves [26]. Although the feedback process encourages self-assessment to improve the trainees’ performance, however, some of those performing well may underestimate their performance while some of those underperforming may overestimate themselves [14,27-28]. Over time, regular self-assessment of trainees may help them recognize the difference between the self-assessment and the external assessment to avoid over or underestimation of their abilities [11].

Whenever face-to-face feedback is reduced, the learners should be encouraged to self-review their progress. Self-review is a fantastic learning tool allowing the trainees to monitor their performance in a way that reduces their dependence on their trainers allowing them to grow their self-confidence and become more mature clinicians [4]. This is potentially a future-proof technique safeguarding their training progression should social distancing continue.

Feedback models 

Pendleton’s rules form one of the most widely accepted feedback models [25]. In this approach, the positive performance of the trainee is appraised first by the trainee, enabling the creation of a safe environment for discussion. These positive points are then reinforced by the trainer. Consequently, discussing the skills the trainee needs to achieve will be easily accepted. Following this, the trainees are encouraged to suggest what they think could have been done differently. Finally, the trainer suggests alternate models if necessary. Nonetheless, Pendleton’s rules have been criticised for not allowing the trainees the opportunity to reach the goals they have set for themselves. Therefore, it has been considered by some authors/trainers to be a slightly rigid model [29].

Under the current difficult training circumstances, where direct contact is hindered, every effort should be made to help all trainees. A possible approach, using both direct and facilitative feedback, may be more successful. In this approach, the trainers suggest different educational and clinical learning needs together with giving comments and suggestions to help guide the trainees in their revision and to form a concept of their work [30]. Using the SET-GO method of giving feedback is likely to be helpful in this scenario due to its descriptive and non-judgemental strategy. Also, it is specific and therefore directed towards the behaviour of the trainee. It allows the trainers to direct their trainees to what they think is their strength and weaknesses and allow them to acknowledge them, reflect on them and try to find a solution to the problem [31]. This feedback model can be delivered remotely while allowing the trainee to react to the feedback comfortably in their preferred environment. Furthermore, this strategy avoids embarrassing the trainees when criticised for a particular performance, encouraging them to accept all instructions.

Silverman has invented another model for feedback under the name of an agenda-led, outcome-based analysis (ALOBA) [32]. This model also provides the trainees with a safe environment, reducing the chance for them to be defensive, making their experience more constructive. This is achieved by identifying what the trainees want and allowing them to be actively involved in the whole process. This allows the trainer to concentrate on the goals planned by the trainees. Nevertheless, this model has been criticized for being judgemental [33].

Studies have demonstrated that a structured discussion between the trainer and trainee followed by interactive feedback encourages self-evaluation and allows the trainees to assess their weak areas of training [5-6]. This is also supported by a study known as Prepare to Ask-Discuss-Ask-Plan Together (Prepare to ADAPT). It was suggested that the framework of this approach may help in decreasing the anxiety and stress commonly associated with feedback by making the process clear. Therefore, applying this structure will help improve the relationship between the trainers and their trainees. This technique was found to be easy to use, increasing the number of effective feedback conversations [34]. This can be replicated in online interactions. The problem with this particular model is that it is very time-consuming. In addition, it may prove to be quite difficult for the trainer and the trainee to find a suitable time when they are both following different work schedules. Additionally, it may take longer to discuss a subject that could have been discussed swiftly if the trainer and trainee were sitting together. In this case, self-evaluation and self-reflection could be very beneficial in improving the future performance of a trainee [35]. Trainees comparing their performance to their peers and trainers' instruction could prove to be a beneficial learning tool.

Oeppen RS et al. (2020) suggested the following recommendations to help provide more effective remote feedback: putting trainees at ease by giving them an initial introduction, simplifying the virtual feedback experience by maintaining clear communication, making sure that the trainee understands what has been mentioned in the meeting; if the trainee did not fully understand the feedback, the trainer should repeat the instructions or put it in a different form; when there are several members in the meeting, the process of using the ‘hand-up’ virtual platform feature will be useful, and at the end of the meeting the contents should be summarized verbally and written using bullet points [23]. Mühlbach L et al. (1995) recommended paying attention to the position of the cameras to ensure adequate eye contact in order to help normalize online meetings to help make them feel more like face-to-face meetings [36].

In summary, receiving feedback is one of the most crucial learning tools helping trainees reach their utmost learning potential, accelerating their training progression. The COVID-19 pandemic has had a negative effect on the feedback process, hands-on courses, and regional training programs. Models of feedback should be tailored specifically for different trainees.

## Conclusions

In conclusion, feedback should be descriptive, non-judgemental, detailed and specifically tailored to the trainee’s aims, abilities, and performance. Sticking to one feedback model with all trainees may prove to be too rigid. Adopting a tailored approach to use different feedback models for each trainee is more beneficial than using the same feedback model with all trainees, hence improving their learning curve. Due to the continued instability from the prolonged effects of the COVID-19 pandemic, it is unlikely that there will be a swift return to the traditional method of face-to-face feedback. Therefore, trainers should not forget the lessons they have learned from developing and advancing the existing techniques of remote feedback and continue to use them where a benefit is perceived for their trainees.
